# A Substitute for Finsen Light

**Published:** 1919-06-07

**Authors:** 


### TUBERCULOSIS NOTES.

### A Substitute for Finsen Light.

In the *Ugeskrift for Læger* and the *Hospitalstidende* the Finsen Institute at Copenhagen has issued clinical reports on the results of light treatment of lupus and surgical tubercle. It appears that formerly it was the practice at this institution to employ only local irradiation of the form already well known. Experience has, however, shown that the residuum of refractory cases could be a good deal lessened (*e.g.*, in the case of lupus the percentage of cures rose from 60 per cent. to 80 per cent.) by light baths. These were necessarily mainly artificial, the source of light rays being the carbon arc or mercury-quartz vapour-lamps. Their tests confirm the German teaching that the former of these two types is the more effective. As regards surgical tuberculosis, the sort of case that responds best is the early one; bone and joint (but not spinal caries), and testicular and peritoneal affections are mentioned as being suitable. There is even a hint of preference for these general artificial sun baths, to which the whole surface of the body is exposed, over the laborious local irradiation with the Finsen light, with which everyone is familiar. The frequency of lupus of the mucous membranes in Copenhagen is rather excessive, yet Strandberg avers that rhino-laryngological cases may be cured without any local treatment. The conclusion is also that the artificial light can substitute for sunlight, which is good news for northern countries. In the absence of handy water power electric current is generally rather expensive however. In many parts of England, even in an industrial area, the cost of running large carbon arc lamps like those used would be at least seven shillings an hour.

